# Synthetic cationic antimicrobial peptides bind with their hydrophobic parts to drug site II of human serum albumin

**DOI:** 10.1186/1472-6807-14-4

**Published:** 2014-01-23

**Authors:** Annfrid Sivertsen, Johan Isaksson, Hanna-Kirsti S Leiros, Johan Svenson, John-Sigurd Svendsen, Bjørn Olav Brandsdal

**Affiliations:** 1 The Norwegian Structural Biology Centre, Department of Chemistry, Faculty of Science and Technology, University of Tromsø, NO-9037 Tromsø, Norway; 2 Drug Discovery and Design, Department of Chemistry, Faculty of Science and Technology, University of Tromsø, NO-9037 Tromsø, Norway; 3 Department of Chemistry, Faculty of Science and Technology, University of Tromsø, NO-9037 Tromsø, Norway; 4 Centre for Theoretical and Computational Chemistry, Department of Chemistry, Faculty of Science and Technology, University of Tromsø, NO-9037 Tromsø, Norway

**Keywords:** Albumin binding, Drug site II, Isothermal titration calorimetry, Group epitope mapping, Molecular docking, NMR, Crystal structure

## Abstract

**Background:**

Many biologically active compounds bind to plasma transport proteins, and this binding can be either advantageous or disadvantageous from a drug design perspective. Human serum albumin (HSA) is one of the most important transport proteins in the cardiovascular system due to its great binding capacity and high physiological concentration. HSA has a preference for accommodating neutral lipophilic and acidic drug-like ligands, but is also surprisingly able to bind positively charged peptides. Understanding of how short cationic antimicrobial peptides interact with human serum albumin is of importance for developing such compounds into the clinics.

**Results:**

The binding of a selection of short synthetic cationic antimicrobial peptides (CAPs) to human albumin with binding affinities in the μM range is described. Competitive isothermal titration calorimetry (ITC) and NMR WaterLOGSY experiments mapped the binding site of the CAPs to the well-known drug site II within subdomain IIIA of HSA. Thermodynamic and structural analysis revealed that the binding is exclusively driven by interactions with the hydrophobic moieties of the peptides, and is independent of the cationic residues that are vital for antimicrobial activity. Both of the hydrophobic moieties comprising the peptides were detected to interact with drug site II by NMR saturation transfer difference (STD) group epitope mapping (GEM) and INPHARMA experiments. Molecular models of the complexes between the peptides and albumin were constructed using docking experiments, and support the binding hypothesis and confirm the overall binding affinities of the CAPs.

**Conclusions:**

The biophysical and structural characterizations of albumin-peptide complexes reported here provide detailed insight into how albumin can bind short cationic peptides. The hydrophobic elements of the peptides studied here are responsible for the main interaction with HSA. We suggest that albumin binding should be taken into careful consideration in antimicrobial peptide studies, as the systemic distribution can be significantly affected by HSA interactions.

## Background

Human serum albumin (HSA) is the most abundant transport protein present in blood plasma with a normal physiological concentration of 0.6 mM. It is active as a monomer with 585 residues and a molecular weight of 66.5 kDa. HSA has a high overall binding capacity due to a number of diverse binding sites distributed over the whole protein, and binds numerous endogenous and exogenous compounds [1]. The two best characterized binding sites regarding ligand specificity and structural information are drug site I and drug site II located in subdomain IIA and IIIA respectively [2]. HSA is known to have a preference for accommodating neutral lipophilic and acidic drug-like ligands, which corresponds well with its main function as a transporter of fatty acids. Exceptions to this generalization exist, and basic residues have been observed as HSA ligands, although only a few experimental complexes have been published [1]. So far the experimental basic ligand HSA complexes comprise the drug site I fluorescence marker dansyl-L-arginine bound in drug site I, and the anaesthetic compound lidocaine located in a novel binding site [3,4].

HSA acts as a negative acute-phase protein, and its physiological concentration may decrease by as much as a factor of two in a number of physiological and pathological conditions [5]. This fluctuation in HSA plasma level affects the equilibrium between the bound and free fractions of a compound that binds to the transporter protein, and may affect drug dosage strategies [6]. In cases where highly hydrophobic compounds bind to HSA, the overall solubility of the drug in plasma will increase. The increase of drug solubility in plasma is regarded as beneficial, but a high affinity toward HSA will require higher dosages and may be a disadvantage [7]. The dual role of HSA binding depends on compound properties and comparative affinity strengths. Drugs binding to HSA are also prone to alterations caused by allosteric modulations induced by additional drug and fatty acid binding [7]. Changes in concentration of endogenous and exogenous ligands in plasma may further induce release of bound drugs into the free state by competing for the same binding site and result in toxic plasma levels [7].

Antimicrobial peptides (AMPs) are a part of the innate immune system of mammals, insects and plants, and act as a first line of defence against harmful microorganisms [8-11]. Most AMPs share the common features of an overall positive charge and an amphiphilic tertiary structure with clusters of cationic and hydrophobic residues; however, on the sequence and the secondary structure level a broad diversity is observed. AMPs are often the products of a pre-proprotein cleavage that are rapidly and inexpensively produced as an immune response. There are several proposed mechanisms for bactericidal activity of AMPs with interaction and disruption of the bacterial cell membrane being the common trait [12,13]. These unspecific membrane mechanisms are in agreement with experimental observations such as the broad activity and the need for relatively high AMPs concentrations to kill microorganisms. AMPs with receptor-recognition mechanisms exist, but are distinguished by having higher activities and specificities than those that interact with the bacterial membrane [12].

The synthetic cationic antimicrobial peptides (CAPs) studied in this work have been developed based on truncation and systematic mutations of lactoferricin, a natural AMP found in milk [14-16]. The determined pharmacophore established that the minimum motif for antimicrobial activity was two cationic charges and two hydrophobic moieties, and these could be incorporated in sequences as short as di- and tri-peptides [16]. The structure-activity relationship (SAR) has been further studied by incorporation of synthetic hydrophobic moieties and variation of the basic residues [17-20]. Although the peptides are too small to obtain any specific secondary structure, molecular modelling experiments indicate that flexible amphipathic conformations are one of the key properties of CAPs [18]. We have previously found that CAPs bind to albumin in the low μM range, and when HSA was included in cell-based assays at physiological concentrations the minimal inhibitory concentration (MIC) of the CAPs was increased by an order of magnitude [21]. Albumin binding has also recently been reported for other AMPs [22]. Previous ADMET studies on CAPs have mainly focused on protease stability, however blood plasma stability and stability in the main metabolic compartments of the body have also been investigated [20,23-25].

Studies of synthesized lactoferricin as retro, inverso and retroinverso versions support a bacterial membrane-dependent mechanism by being unable to differentiate between any of the activities of the variants [26]. In other investigations incorporating full D-amino acid variants and D-amino acids in the CAP sequences, the antimicrobial activity is still retained compared with the native [16,24]. It is highly unlikely that a receptor-recognition mechanism would be unaffected by any of these structural modifications. Also supporting the membranolytic hypothesis is the fast bacterial killing and reported MIC values in the μM range [24]. Molecular dynamic simulations (MD) and NMR liposome dispersion studies of CAPs in a membrane system provide a reasonable interpretation of possible membrane interaction and indicate cell lysis by the carpet mechanism [27]. The Kallenbach group has studied the effect of scaffold attachment and density enrichment of peptides containing arginine and tryptophan, and suggest that an increase in density enhances antimicrobial activity [28-30].

So far a large amount of work has been done to investigate the different aspects of CAPs, with the main objective to develop a novel class of antibiotics for clinical use. As our previous albumin binding study indicates, HSA reduces the fraction of CAP in plasma when present at physiological concentrations, and hence reduces the antimicrobial activity. This behaviour affects how the CAPs will be managed in the next step of peptide engineering and if it is feasible to aim for systemic distribution in plasma. The small and drug-like molecular structure of the CAPs facilitates in part the potential for further development into new drugs. Plasma protein interaction of drug-like molecules is not uncommon as it is the rule rather than the exception in most cases. Detailed knowledge of the HSA interaction of CAPs would be useful in developing these peptides for use in combating the rise of antibiotic resistant bacteria.

Herein we report the binding of a selection of CAPs to human albumin along with competitive binding experiments with reference ligands for HSA drug site I and II determined by isothermal titration calorimetry (ITC) and NMR water ligand observation with gradient spectroscopy (WaterLOGSY). In order to examine the interaction in more detail NMR experiments applying saturation transfer difference (STD) for group epitope mapping (GEM) and INPHARMA for signal transfer between reference ligand and CAPs were conducted. Molecular docking was also carried out to obtain a better understanding of the binding mechanism.

## Results and discussion

**Figure 1:**
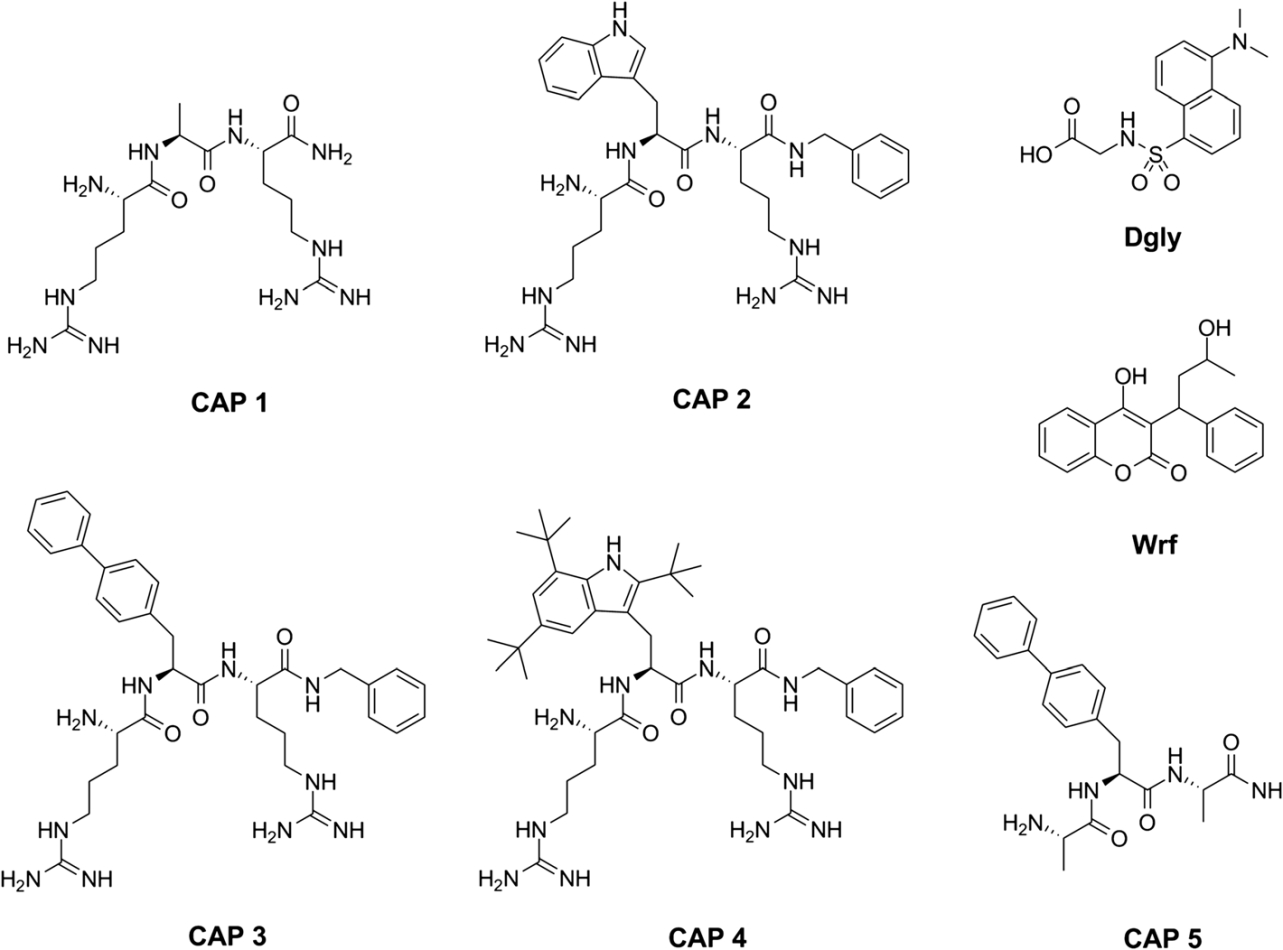
The molecular structure of CAPs and reference ligands used in this study.

We have previously established that several CAPs surprisingly interact with HSA and bind with low μM affinity [21]. However the interaction between the peptides and HSA has not been explored in more detail. In order to examine the binding of CAPs to HSA with respect to drug site I or site II, five peptides were selected consisting of three active CAPs and two inactive control peptides as shown in Figure 1. **CAP 1** and **CAP 5** were included as controls as they contained either the positively charged arginines or one of the hydrophobic moieties necessary for activity [16].

### Antimicrobial activity

The active peptides **CAP 2–4** have been reported elsewhere to have MIC values ranging from 1.3 to 83 μM (Table 1) [21,23,24]. The MIC values of **CAP 1** and **CAP 5** were above the threshold for all of the bacterial strains tested, and they are therefore regarded to be inactive as antimicrobial agents (Table 1). The lack of activity observed for **CAP 1** was assumed to be a consequence of its lack of hydrophobic moieties, thus it does not fulfil the required pharmacophore. The inactivity observed with **CAP 5** can be ascribed to its lack of cationic residues, as it is being composed only of hydrophobic residues.

**Table 1:** Minimal inhibitory concentration values in μM towards selected bacteria

**Peptide**	**Ref.**	***S. aureus***^**a**^	**MRSA**^**b**^	**MRSE**^**c**^	***E. coli***^**d**^	***P. aeruginosa***^**e**^	**GISA**^**f**^
**CAP 1**		>499	>499	-	>499	-	-
**CAP 2**	[23]	83	50	25	-	-	-
**CAP 3**	[21,23]	7.8, 11	11	3	-	-	-
**CAP 4**	[24]	3.2	3.2	1.3	9.7	6.5	3.2
**CAP 5**		>523	>523	-	>523	-	-

^a^*Staphylococcus aureus* strain ATCC 25923.

^b^Methicillin resistant *Staphylococcus aureus* ATCC 33591.

^c^Methicillin resistant *Staphylococcus epidermidis* ATCC 27626.

^d^*Escherichia coli* ATCC 25922.

^e^*Pseudomonas aeruginosa* ATCC 27853.

^f^Glycopeptides intermediate-resistant *Staphylococcus aureus* CCUG 43315.

### Isothermal titration calorimetry

**Figure 2:**
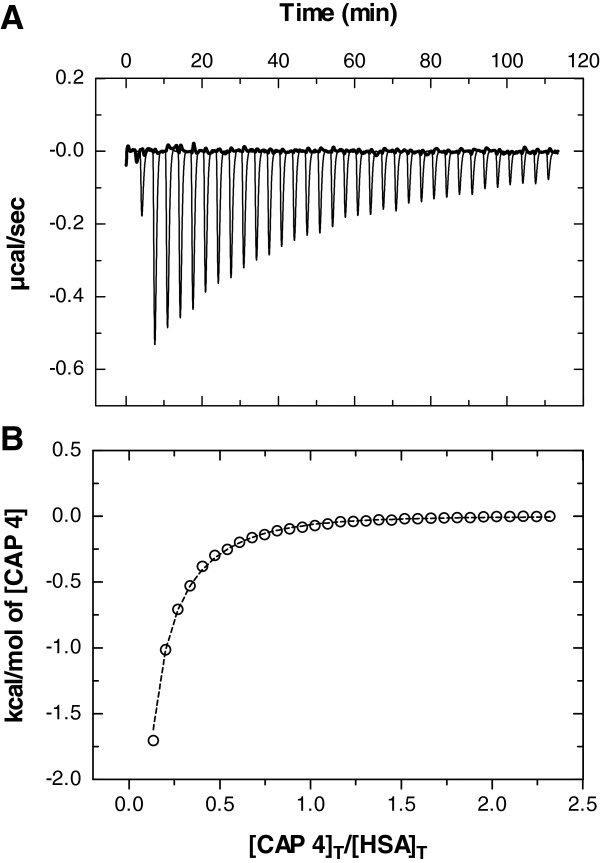
**ITC binding data of CAP 4 titrated into HSA. (A)** Raw data as μcal/sec is plotted against time in min with the control buffer experiments shown in bold line. In **(B)** the fitted independent model is shown as dotted line to isotherm data point presented as open circles. The first data point is omitted from the analysis. The data was obtained with the CSC 5300 Nano-Isothermal Titration Calorimeter III. Figures made in GrapPad Prism v5.00.

The thermodynamic data for non-competitive ITC experiments are presented in Table 2, and were similar to previously published binding data [21]. The control peptide **CAP 1** did not bind to HSA, whereas the active peptides **CAP 2–4** and the control peptide **CAP 5** were found to interact with similar K_d_ values in the low μM range. Raw data along with integrated heats and the binding isotherm model of a typical non-competitive titration experiment is presented in Figure 2 for **CAP 4.**

**Table 2:** Thermodynamic ITC data for CAPs and reference ligands Wrf and Dgly

**Ligand**	**K**_**d**_^**c,†**^	**n**^**d, ‡**^	**ΔG**^**e,§**^	**ΔH**^**f, #**^	**TΔS**^**g, $**^
**CAP 1**	-	-	-	-	-
**CAP 2**^**a**^	23 ± 8	0.67 ± 0.07	-6.32 ± 0.18	-3.7 ± 0.5	2.6 ± 0.7
**CAP 3**^**a**^	22 ± 9 (99 ± 6)^b^	0.77 ± 0.07 (0.36 ± 0.03)^b^	-6.36 ± 0.19 (-5.46 ± 0.04)^b^	-2.8 ± 0.4 (-16.7 ± 1.3)^b^	3.5 ± 0.6 (-11.2 ± 1.4)^b^
**CAP 4**^**a**^	25 ± 9	0.57 ± 0.08	-6.28 ± 0.19	-5.2 ± 1.0	1.1 ± 1.1
**CAP 5**^**b**^	(100 ± 13)	(1.01 ± 0.05)	(-5.46 ± 0.07)	(-3.0 ± 0.2)	(2.5 ± 0.3)
**Wrf**^**b **^**(site I)**	9 ± 4 (28 ± 4)	1.15 ± 0.04 (1.02 ± 0.02)	-6.87 ± 0.19 (-6.21 ± 0.07)	-2.4 ± 0.1 (-3.3 ± 0.1)	4.4 ± 0.3 (2. ± 0.)
**Dgly**^**b **^**(site II)**	11 ± 4 (40 ± 2)	1.08 ± 0.05 (1.16 ± 0.01)	-6.78 ± 0.20 (-6.00 ± 0.02)	-4.5 ± 0.3 (-5.5 ± 0.1)	2.3 ± 0.5 (0.5 ± 0.1)

CSC 5300 Nano-Isothermal Titration Calorimeter III data, values obtained by MicroCal iTC200 presented in parenthesis. (K_d_ in μM, ΔG, ΔH and TΔS in kcal/mol, n denotes the stoichiometric ratio).

^a^Three parallels.

^b^Two parallels.

^c^Error limits taken as the average of the maximal difference between K_d_ and calculated K_d_ ± error limits 95% confidence interval from K_a_ CSC 5300 Nano-Isothermal Titration Calorimeter III, default error calculations based on least square fit and *χ*^2^ with iTC_200_.

^d^Error limits are the averages of ± 95% confidence interval of the parallels CSC 5300 Nano-Isothermal Titration Calorimeter III, default error calculations based on least square fit and *χ*^2^ with iTC_200_.

^e^Error limits taken as the average of the maximal difference between ΔG and calculated of ΔG ± error limits 95% confidence interval from K_a_ CSC 5300 Nano-Isothermal Titration Calorimeter III, default error calculations based on least square fit and *χ*^2^ with iTC_200_.

^f^ Error limits are the averages of ± 95% confidence interval of the parallels CSC 5300 Nano-Isothermal Titration Calorimeter III, default error calculations based on least square fit and *χ*^2^ with iTC_200_.

^g^Error limits by cumulative addition of error limits of ΔG and ΔH.

^†^Deviation between parallels < 16%, except Wrf with 39%. ^‡^ Deviation between parallels < 21%. ^§^ Deviation between parallels < 3%. ^#^ Deviation between parallels < 12%. ^$^Deviation between parallels < 20%, except CAP 4 with 63%. The symbol “-“denotes no measured binding.

The binding constants K_d_ for **CAP 2–5** were in the same μM range 22–25 μM (99–100 μM) (data obtained with MicroCal iTC_200_ are shown in parenthesis), indicating that all of the CAPs bind with the same affinity towards HSA (Table 2). The binding profiles show that there was both a favourable negative enthalpic and a positive entropic contribution to the free energy. For the known site I reference ligand Wrf (warfarin) the binding strength and thermodynamic profile were found to be similar compared with previous published studies, with our K_d_ 9 μM (28 μM) slightly higher compared with the previously reported values 2.9–3.8 μM [31-33]. The binding affinity for drug site II reference ligand Dgly (dansylglycine) of 11 μM (40 μM) is also slightly higher than the reported 1.7–3.2 published in other studies [34,35]. The corresponding free energies for Wrf and Dgly were respectively -6.9 (-6.2) and -6.8 (-6.0) kcal/mol, with both favourable enthalpic and entropic contributions. By comparison, the CAPs binds with K_d_ in the range 22–25 μM (99–100 μM), corresponding to free energies of -6.3 to -6.4 kcal/mol (-5.5 kcal/mol). When comparing the data obtained with the two instruments, the MicroCal iTC_200_ and the CSC 5300 Nano-Isothermal Titration Calorimeter III, the data values were observed to contain a variation. But there were consistency within the deviations in the binding affinities for the ligands determined with both instruments, hence if comparing the K_d_ obtained with the MicroCal iTC_200_ data the values showed 3–4 times lower affinities compared with the CSC 5300 Nano-Isothermal Titration Calorimeter III, *e.g.* 99 μM compared with 22 μM for **CAP 3**, 28 μM and 9 μM for Wrf and 40 μM and 11 μM for Dgly. Hence comparing the **CAP 3** and **CAP 5** binding in Table 2, **CAP 5** was considered to bind in the same affinity range as the rest of the CAPs. The major difference in the comparison of the data between the instruments was the enthalpy-entropy profile of **CAP 3** that changed to high favourable enthalpy but unfavourable entropy contribution with the MicroCal iTC_200_. The stoichiometry of the reference ligands Wrf and Dgly and the control peptide **CAP 5** revealed a 1:1 ratio. Whereas the other peptides, **CAP 2–4**, the stoichiometry decreased to 0.6-0.8 for the CSC 5300 Nano-Isothermal Titration Calorimeter III instrument data, and even lower (0.4) for **CAP 3** in the MicroCal iTC_200_ obtained data. The control buffer experiments did not show any significant heat of dilution signal for any of the experiments.

### Competitive binding in drug site II

**Figure 3:**
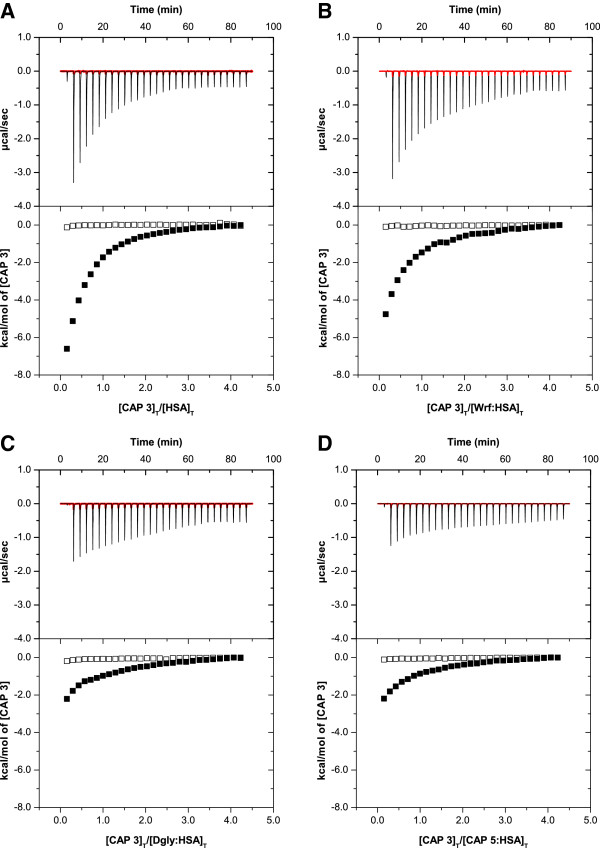
**ITC raw data and integrated heats of CAP 3 titration into HSA. (A)** CAP 3 titrated into HSA solution (reference data) and **(B)** competitive experiments of HSA incubated 1:3 molar ratio with drug site I reference ligand Wrf, **(C)** with drug site II reference ligand Dgly and **(D)** with control peptide **CAP 5**. Control buffer titration is shown in red line in the upper panel and open squares in the lower panel. (The molar ratio in the buffer control was set to the same as the protein ligand ratio merely for the purpose of interpretation). Data collected with MicroCal iTC_200_. Figures made in Origin® 7.0.

To further try to identify which of the numerous binding sites of HSA the CAPs were interacting with, competitive ITC experiments with the reference ligands Wrf and Dgly were conducted. Wrf only interacts with drug site I, whereas Dgly has a secondary binding site in drug site I in addition to its primary site in drug site II (Additional file 1: Figure S1). The competitive ITC results showed that the peptides competed with the drug site II reference ligand Dgly, and not with drug site I ligand Wrf. Competition was indicated by a significant decrease in the heat signals in all experiments where **CAP 2**, **CAP 3**, and **CAP 4** were titrated into HSA incubated in 1:1 molar ratio with either Wrf or Dgly (data not shown). More pronounced was the decrease seen in the signals when HSA was incubated with 1:3 molar ratio either with Dgly or control peptide **CAP 5**, as shown in Figure 3 for **CAP 3** titrations. The competitive effects for **CAP 3** were equally strong for Dgly and **CAP 5**. Similar results were obtained for **CAP 5** titrations into HSA incubated with Wrf or Dgly in molar ratio 1:3, data provided in Additional file 1: Figure S2. Control experiments reversing the titration order of the reference ligands and CAPs were performed for selected peptides and showed the same trends in competition pattern, data provided in Additional file 1: Figures S3 and S4. None of the control experiments with buffer in the cell indicated any significant ligand-ligand interactions in the competitive experiments. It should be noted that the competition experiments between Wrf and the peptides might indicate partial displacement of the ligand incubated with HSA. However, a more likely explanation is the alteration of the free ligand concentration due to peptide-Wrf interactions in solution. We see some interaction between Wrf and peptides in the NMR experiments (see below), which possibly lower the free concentration of the peptide in the ITC competition experiments. Nonetheless, the ITC data is clear on the main binding site as is evident from the Dgly competition experiments.

**Figure 4:**
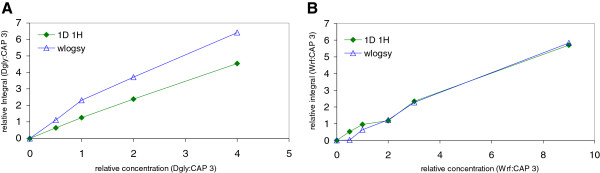
**Competetive WaterLOGSY between CAP 3 and Dgly (A) and Wrf (B). (A)** shows how Dgly perturbs 1:1 linear buildup of the WaterLOGSY effect which is proportional to protein binding, in **CAP 3** relative to the concentration of added Dgly, whereas **(B)** shows that Wrf does not produce the same effect. Ligand:protein ratio was 20:1.

As a complementary technique, NMR was used to probe binding to HSA with WaterLOGSY experiments [36]. Weak binding could be confirmed for **CAP 3** and **CAP 5**, but not for **CAP 1** (Additional file 2: Figures S5 and S6). The **CAP 3** peptide was selected as a representative CAP for competitive binding experiments in WaterLOGSY experiments versus Wrf and Dgly. The WaterLOGSY response to addition of competitive ligand shows a clear reduction in **CAP 3** WaterLOGSY when titrated with Dgly but not with Wrf (Figure 4), thus confirming the ITC finding that **CAP 3** and Dgly bind to the same site on HSA. There were no indications of any direct interactions between Dgly and the studied CAPs before the addition of HSA to the sample. However, a weak direct interaction between **CAP 3** and Wrf in solution was observed as weak direct NOE correlations (also inverting the NOE sign to negative) between the two ligands in trNOE experiments as well as a reduction in relaxation times for the biphenyl resonances of **CAP 3** upon Wrf addition (data not shown).

### The CAPs interact with drug site II with their hydrophobic moieties

**Figure 5:**
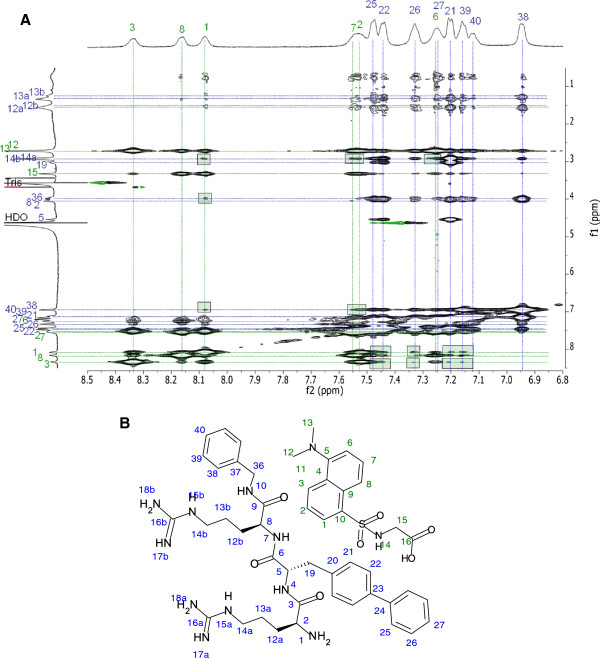
**Protein mediated ligand-ligand contacts. (A)** trNOESY spectra of **CAP 3** versus Dgly acquired using 100 ms mixing time for INPHARMA analysis. Green rectangles indicate groups that transfers signal to each other mediated by the protein. **(B)** Labeling of **CAP 3** and the drug site II reference ligand Dgly in the INPHARMA experiments. The same notation was also used in Table 3.

The INPHARMA results of **CAP 3** and Dgly show signal transfer between the biphenyl residue and the benzyl capping group of the peptide and the reference ligand (see Figure 5 and Table 3). This is best seen in the signal transferred from the tertiary amine methyl groups of Dgly and the hydrophobic moieties of **CAP 3**. The epitope mapping of Dgly is in agreement with the results reported in Lucas *et al.* showing that the strongest interaction is between the N-methyl groups as well as the glycine methylene group of Dgly and HSA [37]. It was clear from the data that the cationic arginines did not contribute to the interaction between **CAP 3** and drug site II of albumin as no signal was transferred to these parts of the peptide.

**Table 3:** NMR-INPHARMA results of CAP 3 versus Dgly

**Entry**		**Dgly**							
	**Label**	**1**	**2**	**3**	**6**	**7**	**8**	**12/13**	**15**
	19	+	-	-	n/a	-	-	+++	++
	21	+	n/a	+	n/a	n/a	++	+++	++
	22	++	n/a	++	n/a	n/a	++	+++	++
	25	++	n/a	++	n/a	n/a	n/a	+++	++
**CAP 3**	26	++	n/a	++	n/a	n/a	-	+++	+
	27	n/a	n/a	n/a	n/a	n/a	n/a	+++	n/a
	36	+	+	-	+	+	-	+++	+
	38	-	-	-	++	++	++	+++	+
	39	+	n/a	+	n/a	n/a	++	+++	++
	40	+	n/a	+	n/a	n/a	++	+++	+

+, ++, and +++ denotes transferred signals and strength between groups in the two ligands (see Figure 5 for labeling), - indicate no signal transferred between groups and n/a denotes not available signals.

**Figure 6:**
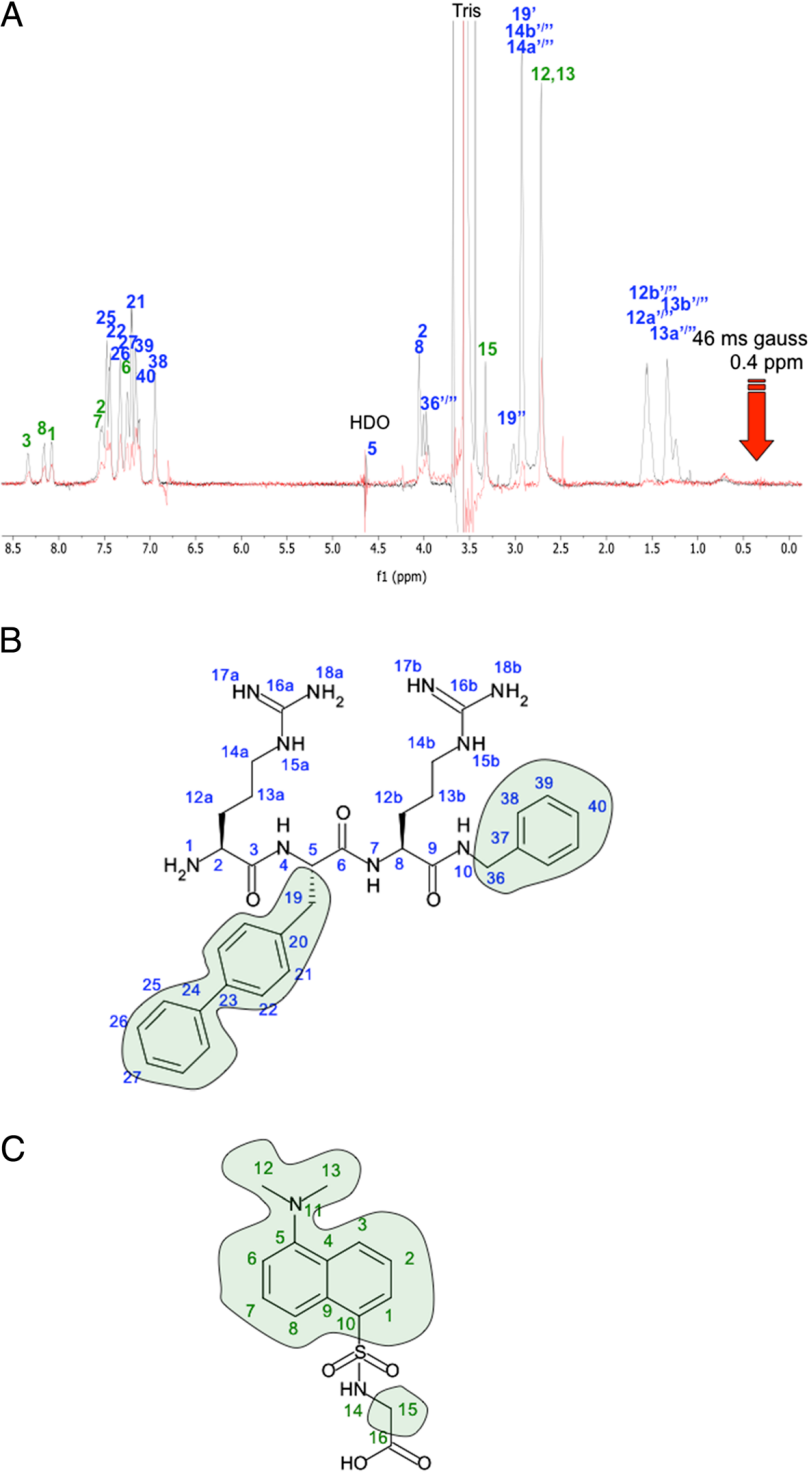
**Protein contact mapping of ligands. (A)** STD spectra of **CAP 3**, Dgly and HSA in 100:1 ratio (red) superimposed on a dpfgse proton spectra acquired with a 10 ms T2 filter for protein suppression (grey). In **(B)** the exited hydrophobic parts of **CAP 3** are indicated in green, and in **(C)** the entire molecule of drug site II reference ligand Dgly was exited.

The saturation transfer difference with group epitope mapping further confirmed the INPHARMA results. The arginines of **CAP 3** were not excited by saturation of protein methyl groups, whereas the hydrophobic parts of the molecules clearly lit up as seen in Figure 6, indicating that they directly take part in interactions with the protein. For Dgly the whole molecule is excited which is in agreement with the INPHARMA results.

### Exploration of modelled binding modes

Drug site II is located in subdomain IIIA of HSA, and has similarities to drug site I in subdomain IIA. Both of the sites are characterized by an apolar pocket and a basic polar patch at the binding site entrance, with a preference for aromatic drug-like ligands with a peripherally negative charge [38,39]. The main difference between the two sites is the packing environment, which enables drug site I to be larger with an enhanced flexibility compared to drug site II [38]. The two fatty acid binding sites (FA) 3 and 4 are also associated with drug site II. In FA3 the methylene tail of the fatty acid is bent into the apolar pocket of drug site II, and in the case of FA4 the carboxyl group of the fatty acid is hydrogen bonded with residues in the polar patch at the site entrance [40,41]. As the HSA ligand preferences for this site are aromatic and negatively charged small ligands, the experimentally determined binding of cationic peptides interacting with this site by both ITC and NMR was unanticipated. The comparably larger size and the multiple positive charges provided by two arginines and the protonated N-terminal of the peptides were expected to exclude CAPs as drug site II ligands.

To further investigate the binding mechanism molecular docking was performed targeting drug site II of HSA. In general, the modelled docking poses and scores supported the experimental findings from both the ITC and NMR data. In all of the docking experiments, the reference drug site II ligand Dgly was docked in a position similar to that occupied by the ligand from original structure, and was located at the top of the docking score ranking list. The modelled conformations of Dgly were found to be virtually identical for all of the target structures, which were accommodated in the apolar pocket of site II. The docking mode was in agreement with the NMR INPHARMA and STD GEM interpretation of Dgly binding. The tertiary amine was buried in the inner hydrophobic part of the site, with the carboxy and sulphate group hydrogen bonding with the polar patch residues at the site entrance. Only docking poses of the CAP library that would compromise the volume occupied by Dgly were accepted, as the results from the competitive ITC data and the NMR WaterLOGSY indicate. The reference ligand Wrf for drug site I and the non-binding peptide **CAP 1** were added as negative controls, and were generally given docking scores in the lower part of the ranking list.

The main difference between the docking targets was the conformation of the entrance residue Arg410. This residue is flexible and known to be a highly ligand inducible residue, which is also reflected by high B factors in the structure of 1E78 and 2BXF, or disordered as found for crystal structures 2XW1 and 2XVQ. Three different main conformations were observed, either pointing towards Glu492 as seen in the Prime built and minimized 2XW0 structure, or towards Gln390 (2BXF). An intermediate position was observed for the apo structure 1E78. With Arg410 in 2XVQ resembling the 2XW0 conformation and the side chain in 2XW1 in a similar position as seen in 2BXF. The apo structure 1E78 did not produce satisfactory poses for the system with a few exceptions, and comparing the docking score with the experimental binding data indicated a too weak interaction for these poses. Hence, when the side chain was positioned in the middle of the entrance of drug site II, Arg410 was blocking the binding site. Docking with 2XW0 and 2XVQ as target structures did not produce satisfactory results, as no accepted poses for **CAP 3** or **CAP 4** were obtained. The Arg410 is pointing toward Glu492 in a similar conformation for these structures, which does not seem to be the optimal conformation for CAP binding.

For 2BXF and 2XW1, which had Arg410 conformations toward Gln390, all of the peptides and the reference ligands were satisfactorily docked and with scores in agreement with the experimental ITC data in Table 2. However, 2XW1 produced more numerous and consistent of accepted poses compared to 2BXF for all of the binding CAPs. 2XW1 was also able to rank the non-bonding control peptide **CAP 1** at the bottom of the ranking list. As a consensus for the accepted poses for these targets, the peptides interact with drug site II by one of the hydrophobic moieties, either the indole for **CAP 2** or the Bip side chain for **CAP 3** and **CAP 5** respectively. Interestingly, only the benzyl capping group residue was found to interact with drug site II for **CAP 4**. This is most likely due to the large size of the synthetic Tbt side chain in this peptide. Compared with the NMR INPHARMA results and the epitope mapping by others [37] the signal transferred between the different structural parts of Dgly and **CAP 3** was in agreement with how deep they would be located in the binding site. For instance the signal from the glycine methylene group show indications of transferring to parts of the **CAP 3** which would occupy the outer part of drug site II.

**Figure 7:**
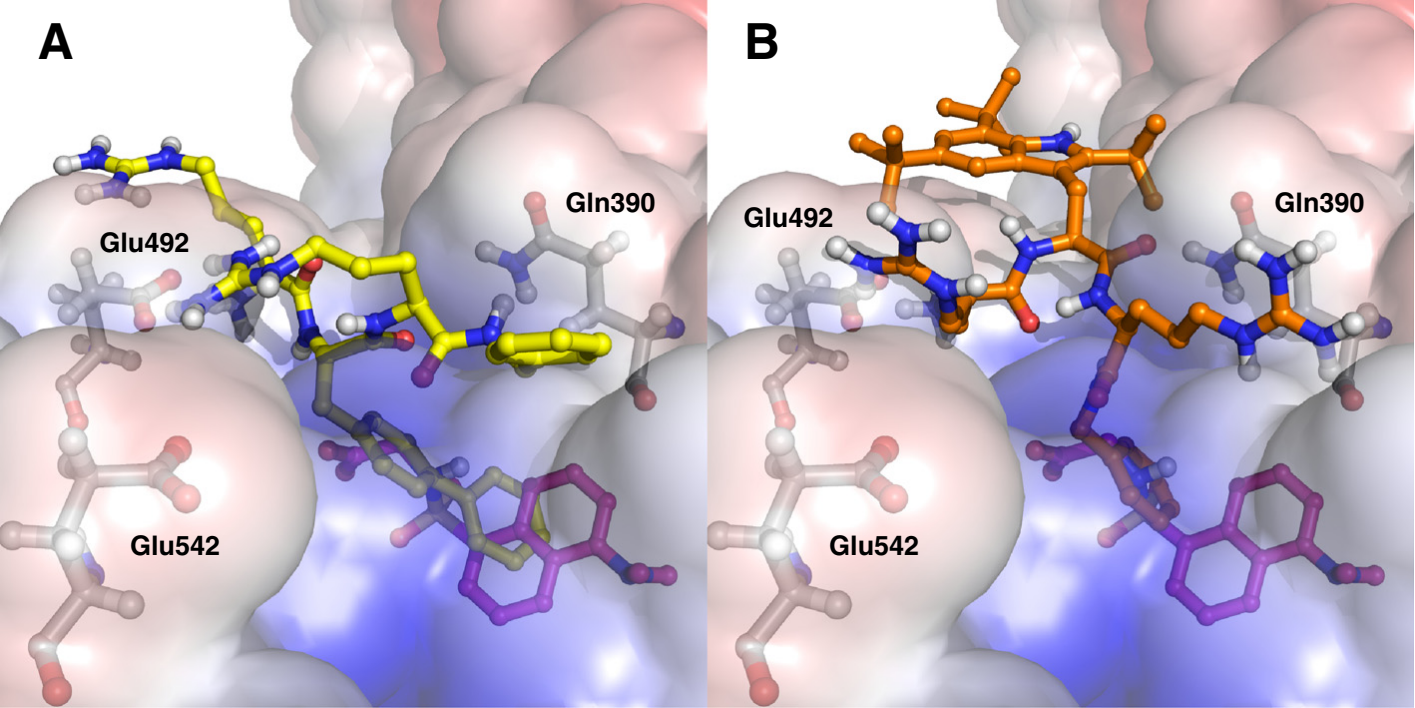
**Molecular models of ligand binding to human albumin obtained with docking. (A)** Docking poses of **CAP 3** in yellow (docking score -8.1 kcal/mol) superimposed on Dgly in magenta (docking score -9.0 kcal/mol) with target 2XW1. **(B)** Docking poses of **CAP 4** in orange (docking score -6.6 kcal/mol) superimposed on Dgly in magenta (docking score -9.0 kcal/mol) with target 2XW1. The calculated electrostatic potential surface of 2XW1 is shown. **CAP 3** is interacting with drug site II with the biphenyl, whereas **CAP 4** is binding with the C-terminal capping benzyl. The lipophilic group in each peptide is comprising the volume occupied by the docked Dgly conformation. Arg410 is omitted in the figures merely for the purpose of clarification, as it would partly cover the bound Dgly and CAPs.

Figure 7 shows docking poses for the highest accepted ranked pose of **CAP 3** and **CAP 4** superimposed on the docked Dgly molecule for target structure 2XW1. The docking scores for the 2XW1 target were in the range of -6.6 to -8.3 kcal/mol for the top ranked accepted pose of the CAPs and -9.0 kcal/mol for Dgly, corresponding to lower μM binding constants. Whereas for the range for 2BXF was -5.7 to -7.7 kcal/mol for the CAPs and -8.0 kcal/mol for the top ranked pose of Dgly. The **CAP 3** was top ranked for both targets with a score of -10.8 (2XW1) and -9.0 (2BXF) kcal/mol which correspond to a binding constant in lower nM region. In these two poses the Bip binds deeper in the binding site than any of the other poses for this peptide, and it is uncertain if it corresponds to a real binding mode when comparing with the experimental binding constant. In general the control peptide **CAP 5** displayed docking poses that resembled the Bip conformation of **CAP 3**. Control peptide **CAP 1** was given docking scores that correspond to mid μM K_d_ values, and docked poses were exclusively located outside drug site II for the targets 2BXF and 2XW1.

From the docking poses, the interaction with drug site II of HSA depends only on the hydrophobic elements of the peptides, and does not involve the cationic residues that are vital for antimicrobial activity. The models are in good agreement with the experimental NMR results in this aspect. This is also evident when comparing the experimental and modelled interaction of the two control peptides **CAP 1** and **CAP 5** with HSA. The control for the cationic part of the pharmacophore, **CAP 1**, does not bind to HSA. Whereas **CAP 5** which is containing only hydrophobic residues interacts with drug site II with similar affinity as the antimicrobial CAPs. The arginines were observed to interact with nearby negatively charged surface residues in the docking poses, but no consistent patterns were observed. The solvent states of the arginine residues are considered equal in the bound and free form of the peptides, and will therefore not contribute to the free energy of the binding. It seems that the CAPs have evaded their own undesirable characteristics as drug site II ligands, as they only interact with one of their lipohilic groups.

Avoiding albumin binding for CAPs would be challenging, as the hydrophobic moieties are crucial for their antimicrobial activity. But observations in the docking poses of **CAP 4** might be a starting point in changing the albumin binding properties. The bulky Tbt side chain of this CAP was observed to be too large to interact satisfactory with drug site II, and hence the interaction was achieved by binding with the C-terminal capping benzyl. If a lipophilic group with similar properties would replace the benzyl, lower albumin binding is anticipated.

### Crystallization

The attempts to crystallize and obtain a complex structure of HSA and CAPs resulted in an apo-structure with fatty acid molecules bound in the seven fatty acid binding sites (Additional file 3), as previously described in the literature [40-42]. In our apo-fatty acid structure we found that for the first time to our knowledge, the direction of the fatty acid bound in FA7 is conclusively determined. The carboxyl group was found to form strong ionic interactions (<3 Å) with the guanidinium group of Arg218, see Additional file 3 Figure S8. Data collection and model refinement statistics are presented in Additional file 3: Table S1.

## Conclusions

In this study we have reported how CAPs interact with HSA within μM affinity by ITC in agreement with previous studies by our group [21]. We have identified the binding site of the peptides to conclusively be drug site II of HSA. The interaction is solely dependent on the hydrophobic moieties present in the peptides. Both NMR experiments and molecular modelling results support that the cationic arginine residues of the peptides do not contribute to the interaction. Since the hydrophobic moieties are an important part of the pharmacophore of CAPs, it will be challenging to design peptides with satisfactory activity with reduced albumin binding properties, and hence it is anticipated that HSA binding of CAPs will be an issue that needs to be addressed in future drug administration strategies.

## Methods

### Ligand library

The molecular structures of the ligands used in this study are presented in Figure 1. The synthesis of **CAP 2 **[23], **CAP 3 **[21,23] and **CAP 4 **[24] have been published elsewhere by our group. The control peptides **CAP 1** and **CAP 5** were purchased from PolyPeptide Laboratories (Strasbourg, France). Reference ligands for drug site I warfarin (A4571) and for drug site II dansylglycine (D0875) were purchased from Sigma. **CAP 2–4** have a common RXR-Bzl scaffold, with X containing a varying hydrophobic side chain. The side groups were indole (Trp) for **CAP 2**, biphenyl (Bip) for **CAP 3** and tri-tert-butyl substituted indole (Tbt) for **CAP 4. CAP 1** and **CAP 5** were included as inactive controls representing the cationic charge or the hydrophilic moiety of the active peptides respectively. Control peptide **CAP 1** contained a methyl group (Ala) as side chain in X that was flanked by arginines and had an amide capped C-terminal. The control peptide **CAP 5** had a Bip in the side group for X flanked by alanines and also contained an amide capped C-terminal. The peptides were chosen for this study based on the diversity in lipophilicity and size of the varying hydrophobic side chain in residue X.

### Microbiological studies

The antibacterial activity of **CAP 1** and **CAP 5** were tested towards *Staphylococcus aureus* strain ATCC 25923, methicillin resistant *Staphylococcus aureus* strain ATCC 33591 and *Escherichia coli* strain ATCC 25922. The studies were performed by Toslab AS employing standard methods [43].

### Isothermal titration calorimetry studies

The experiments were either performed on a CSC 5300 Nano-Isothermal Titration Calorimeter III (Calorimetry Sciences Corporation, Utah, USA) with a cell volume of 1 mL, or a MicroCal™ iTC_200_ with a cell volume of 200 μL (MicroCal, LLC., Northampton, MA, USA). In all experiments lyophilized HSA ~99% essential fatty acid and globulin free (Sigma A3782), was weighed out to a nominal concentration and dissolved prior to the experiments in buffer 50 mM Tris, 10 mM CaCl_2_, pH 7.4 at 25°C. All ligands and peptides were dissolved in the same buffer. Control titrations of ligand into buffer were performed in duplicate to investigate if the heat of dilution would generate a significant signal, and if ligand-ligand interactions were present in the competitive experiments. The same settings as for the corresponding ligand-HSA experiments were applied in the control experiments.

In all experiments applying the CSC 5300 Nano-Isothermal Titration Calorimeter III, the HSA concentration was 0.1 mM and all other peptides and ligand concentrations used were 2.1 mM. In the non-competitive design 33 consecutive 3 μL injections were carried out with 200 s spacing between each injection. A stirring rate of 150 rpm and an isotherm temperature of 25°C were applied. The response signal was measured at 1 s intervals, and a 200 s baseline was collected prior to the first injection for the purpose of assessing the baseline. Non-competitive experiments were carried out either in duplicates or triplicates. In the competitive experimental design HSA was pre-incubated with either the drug site I warfarin or site II dansylglycin ligands, or the peptides **CAP 2–4** in a 1:1 molar ratio. In total 40 consecutive injections were performed with a volume of 5 μL and a spacing of 300 s. The first injection in the series had a dummy volume of 3 μL to account for leakage from the syringe and was omitted in the final analysis. Prior to the first injection, a baseline of 100 s was recorded to ensure instrument stability. The solution was stirred at 150 rpm, and the isothermal temperature set to 25°C. All competitive experiments were carried out in duplicate. For the experiments conducted on the MicroCal™ iTC_200_ instrument the HSA concentration used was 0.21 mM, and the concentration of ligands and peptides were 4.3 mM. In total, 29 injections of 1.35 μL with 180 s spacing between each injection were titrated. The first injection was set to a dummy volume of 0.1 μL due to syringe leakage in the baseline equilibration step. An initial delay of 180 s was set to sample the baseline. The applied reference power was 6 μcal/s and an isothermal temperature of 25°C was used. The solution was stirred at 1000 rpm, applying high feedback mode and a filter period of 5 s. For competitive experiments HSA was pre-incubated with the reference ligand or peptide at a molar ratio of 1:3. For the selected peptides **CAP 3** and **CAP 5** competitive experiments with reversed order of the reference ligands and the CAP were performed as a control of titration order. The same ITC settings were applied for the non-competitive and competitive experiments with the MicroCal™ iTC_200_.

The ITC data was analyzed using the NanoAnalyse software from TA Instruments, v2.0.1 (Waters LLC, New Castle, DE, US) or Origin® 7.0. Independent model fit was used to generate the binding isotherm, and the first injection in all titration experiments was omitted in the binding isotherm analysis. The heat of dilution and unspecific binding signal corresponding to the last injection in each experiment was subtracted from all of the heat signals. The quality of the model fit was examined using the statistical modules of the software, the 95% confidence interval based on a 1000 trial calculations in the NanoAnalyze package, and the default non-weighted least square calculation in Origin® 7.0.

### NMR

All NMR experiments were acquired on an Agilent (Varian) inova spectrometer operating at 599.934 MHz for ^1^H, equipped with a 2^nd^ generation inverse triple resonance HCN cold probe. NMR samples were prepared in 50 mM Tris buffer, 10 mM CaCl_2_, pH 7.4 at 25°C, to final concentrations of 40 μM HSA and 800 μM ligand (Wrf, Dgly, **CAP 1**, **CAP 3** and **CAP 5**). 1D-NOE ePHOGSY for ligand detection via WaterLOGSY [36,44] was acquired as 256 transients, 8 k complex points and 12000 Hz sweep width using 1500 ms mixing time, 1.0 s relaxation delay and a solvent selective pulse of 2.4 ms width at 12 dB. Each ligand addition was recorded with standard ^1^H spectra using watergate (3919) solvent suppression and blank controls were acquired before the addition of HSA samples for STD spectra [45] and transferred NOE (trNOE) spectra for INPHARMA-type analysis [46] were prepared in deuterated buffer to final concentrations of 10 μM HSA and 1 mM ligand (1:100). STD spectra were acquired in 256 transients, 6 k complex data points and 12000 Hz sweep width. Saturation was achieved by 50 cycles of 45.8 ms gaussian shaped saturation pulses centered at 0.4 ppm for the “on” resonance and at 15 ppm for the “off” resonance fid. The difference spectra were produced by internal subtraction and all spectra were acquired with both sculpted solvent suppression during the PFG spin echo and 500 ms solvent presaturation during the relaxation delay. The protein signals were suppressed by a 10 ms T1ρ-spinlock. Finally, the spectra were multiplied with a 3 Hz exponential window function. For reference, double PFG spin echo 1D proton spectra were acquired using the same parameters, including the 10 ms T1ρ-spinlock for protein suppression. NOESY spectra for INPHARMA were acquired in 8 transients as 1440 × 256 data points at mixing times of 50, 100, 200 and 500 ms using zero quantum (ZQ) filter and grad-90-grad randomization. All samples were monitored for line broadening, quickened relaxation and NOE inversion to spot any direct interaction between ligands before the addition of HSA.

### Crystallization studies

An attempt was made to obtain crystals of the human albumin CAP complex for structural determination. Crystallization trials were carried out with recombinant human albumin (rHA) using both the soaking method with fatty acid rHA complex and co-crystallization methods as described by the Curry group [38,40]. Only the soaked fatty acid rHA complex produced crystals of satisfactory diffraction quality. The experimental details for the crystallization of rHA in complex with fatty acid are provided in the Additional file 3.

### Molecular docking

Targets used in the molecular docking experiments were the apo HSA crystal structure 1E78 [47], and the four complex structures 2BXF [38], 2XW1 [3], 2XVQ [3], and 2XW0 [3]. The peptide library was built and prepared in LigPrep, version 2.5 [48], with their stereochemistries retained as all L-residues. All receptor targets were processed using the Protein Preparation Wizard in Maestro, version 9.3 [48]. The docking grids set up in Glide, version 5.8 [48], were centred either on the ligand in the complex structure, or one of the residues belonging to drug site II for the apo structure. A maximum size of ligand diameter and a 14 Å mid point box were utilized. For the docking experiments the standard precision (SP) mode was applied. The grid generation and docking procedure was repeated due to the size difference of the smaller reference ligands and the larger CAPs, applying **CAP 3** as reference ligand for the box size. The ligand library was comprised of the CAPs and the reference ligands Wrf and Dgly presented in Figure 1. For the targets 2XW1 and 2XVQ the side chain coordinates of Arg410 were not reported, and the residue was therefore built and minimized by Prime, version 3.1 [48].

## Abbreviations

ADMET: Administration, distribution, metabolism, excretion and toxicity; AMP: Antimicrobial peptide; Bip: Biphenyl; CAP: Synthetic cationic antimicrobial peptide; Dgly: Dansylglycine; FA: Fatty acid binding site; GEM: Group epitope mapping; HSA: Human serum albumin; INPHARMA: Inter-ligand NOE for pharmacophore mapping; ITC: Isothermal titration calorimetry; MD: Molecular dynamics; MIC: Minimal inhibitory concentration; SAR: Structure-activity relationship; STD: Saturation transfer difference; Tbt: Tri-tert-butyl tryptophan; Wrf: Warfarin; WaterLOGSY: Water ligand observation with gradient spectroscopy.

## Competing interests

The authors declare that they have no competing interests.

## Authors’ contributions

AS carried out the ITC experiments, crystallization, molecular docking and drafted the manuscript. JI carried out of the NMR experiments, and contributed to the data interpretation. HKSL contributed to the data analysis. JS and JSS participated to the conception and in the design of the study. BOB conceived the study, participated in its design and helped to draft the manuscript. All authors read and approved the final manuscript.

## Supplementary Material

Additional file 1 **Competitive ITC binding data. Figure S1.** shows competitive ITC binding data for Wrf and Dgly. **Figure S2.** shows competitive ITC binding data for CAP5 with drug site I ligand Wrf and drug site II ligand Dgly. **Figure S3.** presents the competitive experiments for Wrf with HSA incubated with either CAP3 or CAP5. **Figure S4.** shows competitive experiments for Dgly with HSA incubated with either CAP3 or CAP5. Click here for file

Additional file 2 **WaterLOGSY experiments. Figure S5.** presents WaterLOGSY of HSA and Wrf, Dgly and CAP 1, while **Figure S6.** shows WaterLOGSY of HSA and CAP1, CAP3 and CAP5. Click here for file

Additional file 3 **Crystal structure of albumin-palmitic acid complex.** Crystallization conditions and details of data collection as refinement procedures are presented. A brief discussion of the crystal structure is provided along with data collection and refinement statitistics **(Table S1)**. **Figure S7.** shows the mount crystal, and **Figure S8.** presents the electron density for the fatty acid binding mode of PA 7 in FA site 7. Click here for file
