# Supplementary material for: Synthetic cationic antimicrobial peptides bind with their hydrophobic parts to drug site II of human serum albumin
Source: BMC Struct Biol. 2014 Jan 23;14:4. doi: 10.1186/1472-6807-14-4 (PMC3907362; doi:10.1186/1472-6807-14-4)
Supplement: Additional file 1 — Competitive ITC binding data. Figure S1. shows competitive ITC binding data for Wrf and Dgly. Figure S2. shows competitive ITC binding data for CAP5 with drug site I ligand Wrf and drug site II ligand Dgly. Figure S3. presents the competitive experiments for Wrf with HSA incubated with either CAP3 or CAP5. Figure S4. shows competitive experiments for Dgly with HSA incubated with either CAP3 or CAP5. [file 1472-6807-14-4-S1.docx]

**Figure S1 Competitive ITC binding data of reference ligand Wrf and Dgly. (a)** ITC raw data of Wrf titrated into HSA as reference, and (**b**) into HSA incubated with 1:3 molar ratio drug site II reference ligand Dgly. (**c**) Titration of Dgly into HSA as reference , and (**d**) into HSA incubated with 1:3 molar ratio of drug site I reference ligand Wrf. Control buffer titration experiments are shown in bold red in the uppermost panel and as open squares in the panel below. (The molar ratio in the control experiments is set to the same as the protein peptide experiments merely for the purpose of interpretation.) Data obtained with MicroCal iTC_200_. Figures made in Origin® 7.0.

**Figure S2 Competitive ITC binding data of CAP 5 with drug site I ligand Wrf and drug site II ligand Dgly. (a)** ITC raw data of **CAP 5** titrated into HSA as reference, and (**b**) competitive experiments of HSA incubated with 1:3 molar ratio of drug site I reference ligand Wrf, (**c**) with drug site II reference ligand Dgly. Control buffer titration experiments are shown in bold red in the uppermost panel and as open squares in the panel below. (The molar ratio in the control experiments is set to the same as the protein peptide experiments merely for the purpose of interpretation.) Data obtained with MicroCal iTC_200_. Figures made in Origin® 7.0.

**Figure S3 Competitive ITC binding data of drug site I ligand warfarin. (A)** ITC raw data of Wrf titrated into HSA as reference, (**B**) competitive experiments of Wrf incubated with 1:3 molar ratio of **CAP 3**, and (**C**) **CAP 5**. Control buffer titration experiments are shown in bold red in the uppermost panel and as open squares in the panel below. (The molar ratio in the control experiments is set to the same as the protein peptide experiments merely for the purpose of interpretation.) Data obtained with MicroCal iTC_200_. Figures made in Origin® 7.0.

**Figure S4 Competitive ITC binding data of drug site I ligand dansylglycine. (A)** ITC raw data of Dgly titrated into HSA as reference, (**B**) competitive experiments of Dgly incubated with 1:3 molar ratio of **CAP 3**, and (**C**) **CAP 5**. Control buffer titration experiments are shown in bold red in the uppermost panel and as open squares in the panel below. (The molar ratio in the control experiments is set to the same as the protein peptide experiments merely for the purpose of interpretation.) Data obtained with MicroCal iTC_200_. Figures made in Origin® 7.0.
