# Supplementary material for: Synthetic cationic antimicrobial peptides bind with their hydrophobic parts to drug site II of human serum albumin
Source: BMC Struct Biol. 2014 Jan 23;14:4. doi: 10.1186/1472-6807-14-4 (PMC3907362; doi:10.1186/1472-6807-14-4)
Supplement: Additional file 2 — WaterLOGSY experiments. Figure S5. presents WaterLOGSY of HSA and Wrf, Dgly and CAP 1, while Figure S6. shows WaterLOGSY of HSA and CAP1, CAP3 and CAP5. [file 1472-6807-14-4-S2.docx]

**Figure S5 WaterLOGSY of HSA and Wrf (W), Dgly (D) and CAP 1 (R=RAR) in 1:20 ratio.** Except for the exchangeable NH of **CAP 1**, the aliphatic protons all show an absence of WaterLOGSY effect for **CAP 1**, thus indicating that it does not interact directly with HSA. As expected, the positive controls Wrf and Dgly both display a clear WaterLOGSY effect.

**Figure S6** **WaterLOGSY of HSA and CAP 1 (R=RAR), CAP 3 (B=RBipR) and CAP 5 (A=ABipA) in 1:20 ratio.** All aliphatic protons of **CAP 3** and **CAP 5** except for the quickly rotating methyls of **CAP 5** show a clear WaterLOGSY effect, indicating binding to HSA, whereas **CAP 1** does not.
