# Supplementary material for: Synthetic cationic antimicrobial peptides bind with their hydrophobic parts to drug site II of human serum albumin
Source: BMC Struct Biol. 2014 Jan 23;14:4. doi: 10.1186/1472-6807-14-4 (PMC3907362; doi:10.1186/1472-6807-14-4)
Supplement: Additional file 3 — Crystal structure of albumin-palmitic acid complex. Crystallization conditions and details of data collection as refinement procedures are presented. A brief discussion of the crystal structure is provided along with data collection and refinement statitistics (Table S1). Figure S7. shows the mount crystal, and Figure S8. presents the electron density for the fatty acid binding mode of PA 7 in FA site 7. [file 1472-6807-14-4-S3.docx]

# Crystal structure of albumin-palmitic acid complex with the direction of fatty acid in FA site 7 conclusively determined

## Material & Methods

Recombinant human albumin (rHA) 200 mg/ml from Novozymes Biopharma UK Ltd was gelfiltrated on a Superdex200 column with buffer composition 50 mM KH_2_PO_4_, 150 mM NaCl pH 7.5. Monomer fractions were pooled and up-concentrated before saturated with palmitic acid (PA), C16:0, dissolved in slightly heated 20 mM KH_2_PO_4_ pH 7.5 buffer as described in Curry *et al* [[1](#_ENREF_1)]. In short, 100 μL 155 mg/ml rHA was added to 960 μL 2.6 mM nominal PA solution and incubated at room temperature for 1 hour. Excess PA was removed by centrifugation and the HSA:PA complex concentrated by an iterative wash cycles applying 10 kDa filter (Amicon Millipore). Concentrated HSA, ca 100 μL were diluted with 450 μL 20 mM KH_2_PO_4_ pH 7.5 buffer containing nominal 0.1 mM PA before repeatedly being concentrated. After three cycles the samples were concentrated to approximate 100 mg/mL prior to crystallization experiments. Sitting drops of 3.5 μL protein + 3.5 μL reservoir solution and a reservoir size of 1 mL were equilibrated at 4 °C for 2 days before streak seeding. Crystallization conditions were 26% PEG 3350, 50 mM KH_2_PO_4_ pH 7.2 and protein concentration 120 mg/ml in 20 mM KH_2_PO_4_ at pH 7.5 containing nominal 0.1 mM PA. Crystals were harvested in capillary tubes with gentle soaking conditions, 30% PEG 3350, pH 7.2, 50 mM KH_2_PO_4_ and ligand concentration 3 mM RARNH_2_ for 15-20 minutes. The data was collected on an in-house rotating anode with Kα wavelength 1.5418 Å at room temperature. The collection strategy was 0.3° oscillation per frame, totally 240 degrees of data, 190 mm detector to crystal distance and 25 s exposure per frame. The data was integrated by XDS [[2](#_ENREF_2)] and scaled with SCALA [[3](#_ENREF_3)] in the CCP4 package [[4](#_ENREF_4)]. The structure was solved by molecular replacement applying a rHA:PA complex as template, PDB entry code 1E7H [[5](#_ENREF_5)]. The model was refined by iterative steps of model building in Coot [[6](#_ENREF_6)] and refinement by REFMAC [[7](#_ENREF_7)]. The B-factors were calculated by Baverage [[4](#_ENREF_4)], and the structure was assessed by Procheck [[8](#_ENREF_8)].

## Results & Discussion

The structure of rHA in complex with PA was solved to a resolution of 2.35 Å without any peptide bound. In Figure S5 the crystal from which the dataset was collected from is viewed 90° apart in a) and b) with an estimated crystal size of 0.4x0.6x0.6 mm^3^. The cell was monoclinic C2, with cell parameters a=189.37 Å, b=39.00 Å and c=96.30 Å and β=105.48°. The statistics for the data collection and refinement is presented in Table S1. Overall the structure resembled other albumin fatty acid complexes for medium and long fatty acids [[1](#_ENREF_1), [5](#_ENREF_5), [9](#_ENREF_9)]. The main difference observed between our structure and previously published ones was the fatty acid found bound in site 7 (FA7), where for the first time to our knowledge the direction of the fatty acid in FA7 is determined. The PA bound in FA7 was clearly seen to interact with its carboxyl group with residue Arg218 as shown in Figure S6. The hydrogen bonds between the carboxyl group and the guanidinium group of Arg218 were measured to be 2.76 Å, 2.80 Å and 3.12 Å, respectively. The density of the first 14 carbons in the methylene tail was clearly defined, and the overall conformation of the methylene tail was similar to previously reported fatty acid conformations. Residue Arg257 located in the other end of the fatty acid binding site is therefore not involved in the carboxyl head group interaction as previously discussed by others [[5](#_ENREF_5)].


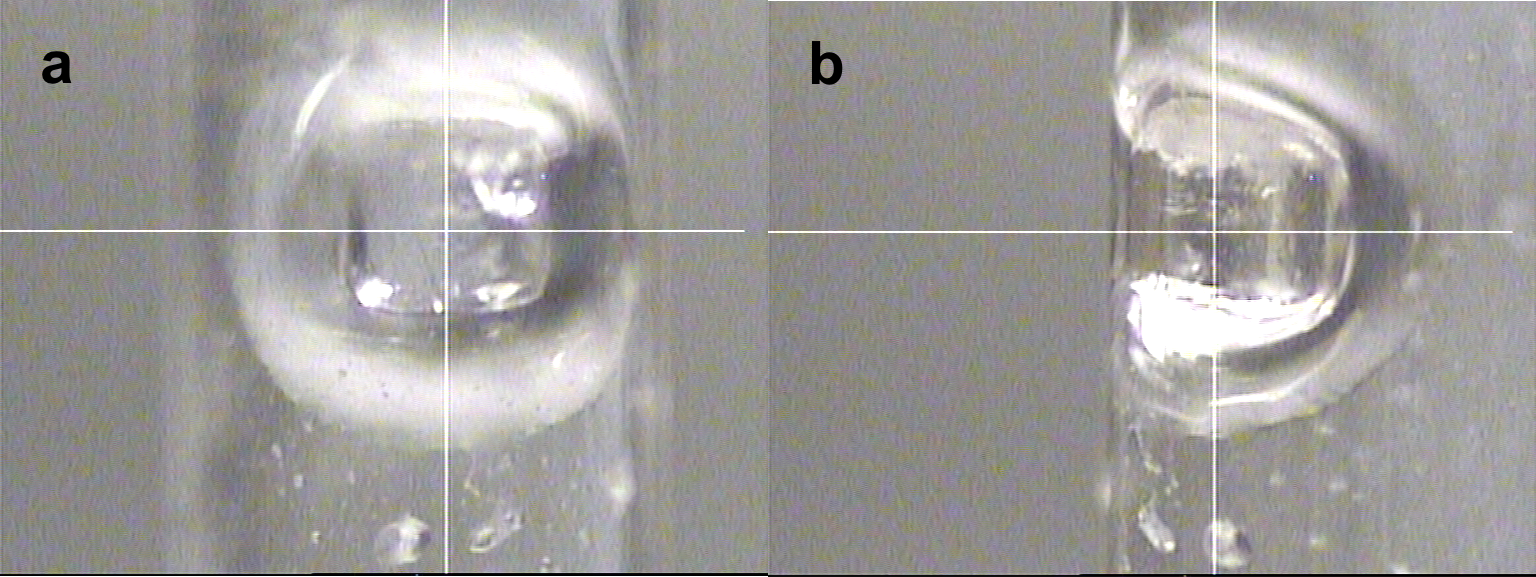


**Figure S7:** The rHA:PA crystal mounted in a capillary tube seen a) from the front and b) side (a 90° rotation from panel a) with an estimated crystal dimension of 0.4x0.6x0.6 mm^3^.

***
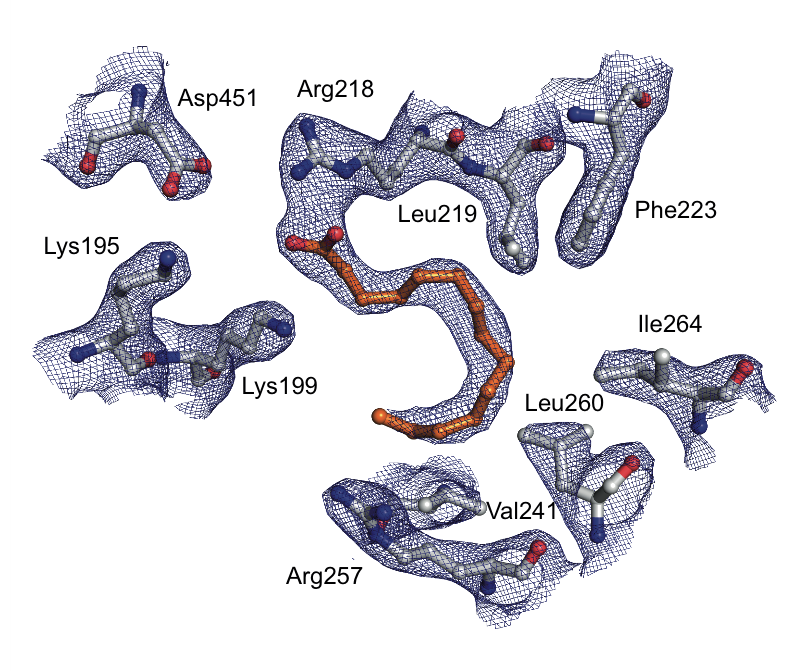
***

**Figure S8:** The fatty acid binding mode of PA in site 7 is conclusively determined to interact with the carboxyl group with hydrogen bonds to Arg218 measured to be 2.76 Å, 2.80 Å and 3.12 Å. The sigma level of the 2Fo-Fc electron density map is set to 1.0σ.

Comparing our structure and the previously reported rHA:PA complex (1E7H), there were observed some minor variations in the interactions between the carboxyl groups of the fatty acid in several of the fatty acid binding sites. This indicates that the interaction between the fatty acid head groups and the side chains in the binding sites inherent some flexibility. The complex structure with fatty acid with carbon length 12 (1e7f), C12:0, has an Arg218 side chain conformation which is overlapping with the carboxyl group in FA7 found in our structure [[5](#_ENREF_5)]. Another observation in the previously published rHA:PA complex (1E7H) is the presence of a water molecule hydrogen binding to Arg218 where the carboxylic group is defined in our structure. This might be interpreted as electron density traces of the fatty acid head group in view of our finding.

**Table S1:** Data collection and model refinement statistics of rHA:PA complex, pdb entry 4BKE.

| **Data collection** |  |
| --- | --- |
| X-ray source | In-house rotating anode |
| Space group | *C21* |
| Unit cell parameters (Å, °) | a=189.37  b=39.00  c=96.30  *β*=105.48 |
| Resolution (Å) (highest bin) | 38.14-2.35 (2.48-2.35) |
| Wavelength (Å) | 1.5418 |
| Total no of reflections | 140 265 (19 999) |
| No of unique reflections | 27 926 (3 981) |
| Multiplicity | 5.0 (5.0) |
| Completeness (%) | 96.5 (95.2) |
| Mean (<I/<σI>) | 9.3 (2.2) |
| R_merge_^†^ | 0.066 (0.664) |
| R_p.i.m._^‡^ (*I^+^* & *I^-^*) | 0.037 (0.364) |
| **Model refinement** |  |
| PDB entry |  |
| Resolution (Å) | 20-2.35 |
| R_work_ | 19.75 |
| R_free_^§^ | 25.43 |
| No of protein residues | 582 |
| No of atoms | 4608 |
| No of water molecules | 26 |
| Ligands | 7 |
| rmsd from ideal bond length (%) | 0.009 |
| rmsd from ideal bond angles (°) | 1.125 |
| Average B-factor |  |
| Main chain | 59.19 |
| Side chain | 58.57 |
| Water | 49.03 |
| rmsd in B-factors main/side chain | 0.457/2.003 |
| Ramachandran plot (Procheck) |  |
| Favoured regions | 93.9 % (512) |
| Additional allowed regions | 5.1 % (28) |
| Generously allowed regions | 0.7 % (4) |
| Disallowed regions | 0.2 % (1) |

*^†^ R_merge_* Σ_hkl_Σ_i_|I_i_(hkl)-〈I(hkl)〉| Σ_hkl_ Σ_i_I_i_(hkl*)*, where *I(hkl)* is the intensity of reflection *hkl*, *Σ_hkl_* is the sum over all reflections and *Σ_i_* is the sum over *i* measurements of reflection *hkl.*

^‡^ R_p.i.m._ Σ_hkl_[1/(N-1)]^1/2^Σ_i_|I_i_(hkl)-〈I(hkl)〉| Σ_hkl_ Σ_i_I_i_(hkl) *)*, where *I(hkl)* is the intensity of reflection *hkl*, *Σ_hkl_* is the sum over all reflections and *Σ_i_* is the sum over *i* measurements of reflection *hkl.*

^§^ R_free_ is the R_work_calculated using randomly 7.2 % of the data set omitted from the refinement.

## References

1. Curry S, Mandelkow H, Brick P, Franks N: **Crystal structure of human serum albumin complexed with fatty acid reveals an asymmetric distribution of binding sites**. *Nat Struct Biol* 1998, **5**(9):827-835.

2. Kabsch W: **XDS**. *Acta Crystallogr D Biol Crystallogr* 2010, **66**(Pt 2):125-132.

3. Evans P: **Scaling and assessment of data quality**. *Acta Crystallogr D Biol Crystallogr* 2006, **62**(Pt 1):72-82.

4. Winn MD, Ballard CC, Cowtan KD, Dodson EJ, Emsley P, Evans PR, Keegan RM, Krissinel EB, Leslie AGW, McCoy A *et al*: **Overview of the CCP4 suite and current developments**. *Acta Crystallogr D Biol Crystallogr* 2011, **67**(Pt 4):235-242.

5. Bhattacharya AA, Grüne T, Curry S: **Crystallographic analysis reveals common modes of binding of medium and long-chain fatty acids to human serum albumin**. In: *J Mol Biol.* vol. 303; 2000: 721-732.

6. Emsley P, Lohkamp B, Scott WG, Cowtan K: **Features and development of Coot**. *Acta Crystallogr D Biol Crystallogr* 2010, **66**(Pt 4):486-501.

7. Vagin AA, Steiner RA, Lebedev AA, Potterton L, McNicholas S, Long F, Murshudov GN: **REFMAC5 dictionary: organization of prior chemical knowledge and guidelines for its use**. *Acta Crystallogr D Biol Crystallogr* 2004, **60**(Pt 12 Pt 1):2184-2195.

8. Laskowski R, MacArthur MW, Moss DS, Thornton J: **PROCHECK: a program to check the stereochemical quality of protein structures**. *Journal of Applied Crystallography* 1993, **26**:283-291.

9. Petitpas I, Grüne T, Bhattacharya AA, Curry S: **Crystal structures of human serum albumin complexed with monounsaturated and polyunsaturated fatty acids**. *J Mol Biol* 2001, **314**(5):955-960.
